# *Listeria monocytogenes* folate metabolism is required to generate N-formylmethionine during infection

**DOI:** 10.1128/mbio.01385-23

**Published:** 2023-09-14

**Authors:** Qing Tang, Michelle L. Reniere

**Affiliations:** 1 Department of Microbiology, School of Medicine, University of Washington, Seattle, Washington, USA; University of Illinois Chicago, Chicago, Illinois, USA

**Keywords:** virulence, translational control, folate metabolism, *Listeria monocytogenes*

## Abstract

Folic acid and its derivatives are required for the synthesis of purines, pyrimidines, and some amino acids. Antifolate antibiotics that target the folic acid metabolism pathway are commonly used for the treatment of listeriosis caused by the intracellular pathogen *Listeria monocytogenes* (*Lm*). In recent work in *mBio*, Feng et al. sought to understand the role of folic acid metabolism in *Lm* virulence (Y. Feng, S. Chang, D. A. Portnoy, 2023, mBio https://doi.org/10.1128/mbio.01074-23). The authors discovered that N-formylmethionine, an amino acid utilized by bacteria to initiate protein synthesis, is crucial for *Lm* intracellular growth and pathogenesis. Surprisingly, purines and thymidine were found to be dispensable for *Lm* infection. Together these results demonstrate that while *Lm* can obtain many essential nutrients from the host cytosol, including purines and most amino acids, it requires N-formylmethionine biosynthesis to properly regulate translation initiation during infection.

## COMMENTARY

**Fig 1:**
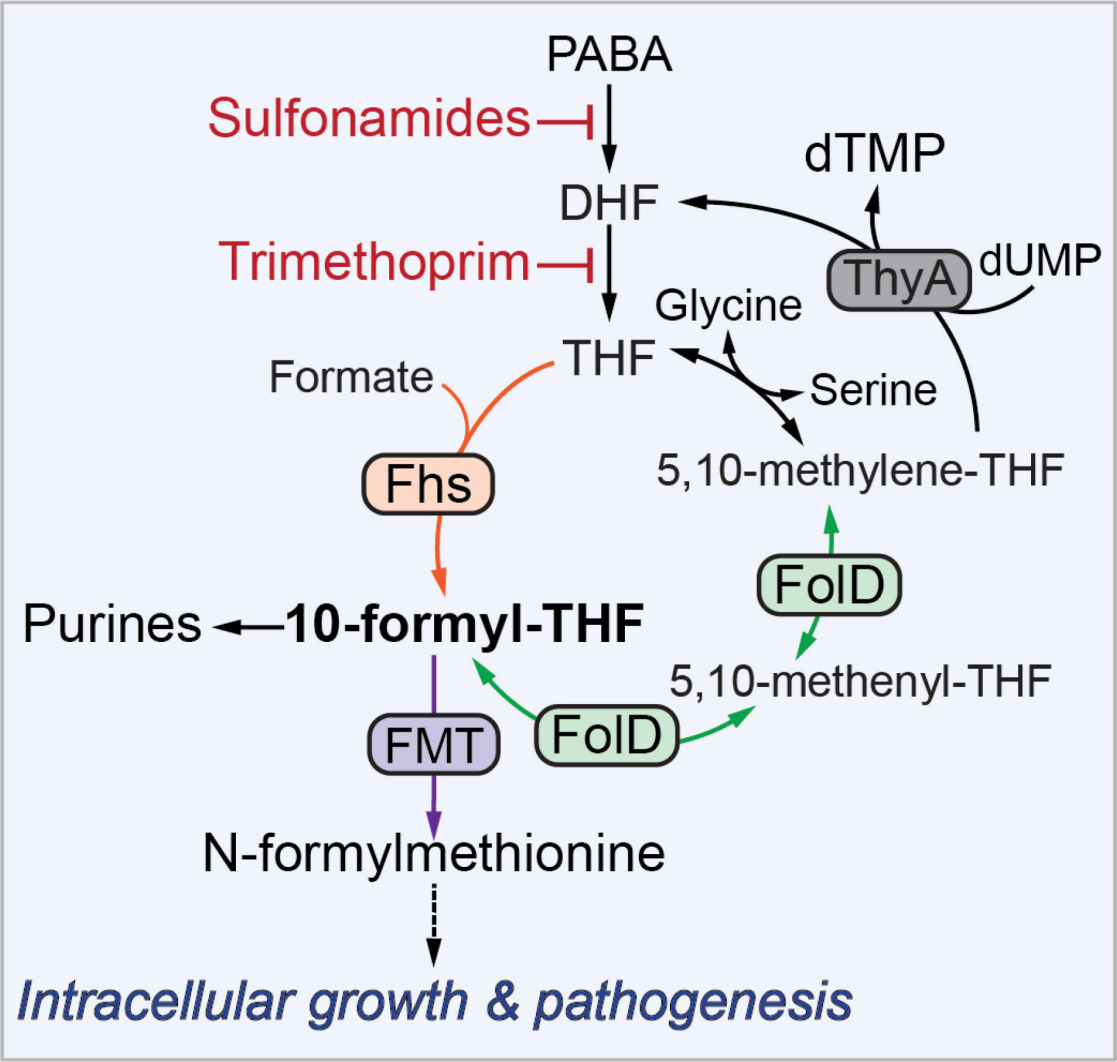
Folate metabolism in *Listeria monocytogenes*. FolD and Fhs both produce 10-formyl-THF, a critical precursor for the biosynthesis of purines, N-formylmethionine, and deoxythymidine monophosphate (dTMP). In recent work, Feng et al. investigate which folate-derived metabolites are critical for *L. monocytogenes* virulence (1). PABA, para-aminobenzoic acid; DHF, dihydrofolate; THF, tetrahydrofolate; ThyA, thymidylate synthase; Fhs, formyltetrahydrofolate synthetase; FMT, methionyl-tRNA formyltransferase; FolD, bifunctional methylenetetrahydrofolate dehydrogenase/methenyltetrahydrofolate cyclohydrolase.

Folic acid and its derivatives (folates) are crucial components of one-carbon metabolism, which is essential for the synthesis of purines, pyrimidines, and some amino acids. Antifolate antibiotics, such as sulfonamides and trimethoprim, block tetrahydrofolate (THF) synthesis and are commonly used to treat bacterial infections, particularly in patients intolerant to β-lactam antibiotics. However, the specific functions of folate metabolism during infection by intracellular pathogens such as *Listeria monocytogenes* (*Lm*) are not well understood. Although *Lm* can obtain many nutrients from the host cytosol, it is not able to import folic acid or its metabolites, rendering the folate biosynthesis pathway critical for bacterial replication *in vivo*. In recent work in *mBio*, Feng et al. aimed to identify the specific folate-derived metabolites required for *Lm* infection (1). This study revealed that while the synthesis of purines and thymidine was dispensable for *Lm* pathogenesis, N-formylmethionine is crucial for the intracellular growth and virulence of *Lm* (Fig. 1).

*Lm* is a facultative intracellular pathogen capable of invading and replicating within both phagocytic and non-phagocytic cells. After invading host cells, *Lm* disrupts its internalization vacuole and translocates to the cytoplasm where it replicates. *Lm* then utilizes actin-based motility to move within the cytoplasm and spread from cell to cell without being exposed to the extracellular environment. Previous research conducted by the Portnoy laboratory established that synthesis of the THF precursor, para-aminobenzoic acid, is upregulated during infection and is necessary for vacuolar escape, cytosolic replication, and actin-based intercellular spread (2). This finding suggests that THF and its derivatives, such as 10-formyl-THF, play a critical role in the virulence of *Lm*. In this study, Feng et al. demonstrate that *Lm* encodes two enzymes that produce 10-formyl-THF. FolD is the primary enzyme responsible for synthesizing 10-formyl-THF during infection, while Fhs plays a measurable role only in the absence of FolD (Fig. 1). These results are consistent with the fact that Fhs uses the fermentative metabolite formate as a substrate and therefore may only be active *in vivo* during growth in low-oxygen environments. Disrupting synthesis of 10-formyl-THF by simultaneous deletion of *folD* and *fhs (∆fhs folD::Tn*) resulted in significant defects in intracellular growth and virulence to similar levels as a mutant that lacks folate synthesis (∆*pabBC*).

As depicted in Fig. 1, the *Lm* folate pathway is required for the biosynthesis of deoxythymidine monophosphate (dTMP), glycine, serine, purines, and formylated methionine (N-formylmethionine[fMet]). It was previously established that *Lm* obtains glycine and serine from the host cytosol (3); therefore, synthesis of these amino acids is not required *in vivo*. Feng et al. demonstrate that while thymidylate synthase is essential for bacterial growth *in vitro* and in macrophages, dTMP derived from the folate pathway is dispensable for *Lm* infection in mice, consistent with previous observations in *Staphylococcus aureus* (4). Additionally, they found that disruption of the gene encoding the repressor of purine biosynthesis PurR rescued the ∆*folD* defects in intracellular growth and intercellular spread. These results suggested that increasing purine synthesis in the ∆*folD* strain would restore virulence. However, neither disruption of *purR* nor exogenous purine supplementation rescued the intracellular growth or pathogenesis of the ∆*fhs folD::Tn* mutant. Furthermore, a purine auxotrophic strain exhibited only a moderate virulence defect. These findings demonstrate that purine insufficiency is not the primary cause of the intracellular growth defect of the 10-formyl-THF-deficient mutant and further suggests that *Lm* can obtain purines and thymidine from the host environment.

In addition to purine and pyrimidine synthesis, 10-formyl-THF is a one-carbon unit donor for fMet biosynthesis via the formyltransferase FMT (Fig. 1). While not required for prokaryotic translation, the formylated initiator tRNA (fMet-tRNA^i^) increases the efficiency and fidelity of translation initiation. Feng et al. establish that the 10-formyl-THF-deficient mutant lacks fMet and exhibits growth defects comparable to a ∆*fmt* mutant, with modest growth delays in broth and in macrophages, but final densities similar to wild-type *Lm*. However, both *Lm* mutants lacking fMet were modestly attenuated in the spleens and dramatically attenuated in the livers of infected mice, demonstrating that while fMet is not strictly required for *Lm* replication, it is critical for *Lm* pathogenesis. These data further suggest that although *Lm* can obtain most amino acids from host cell cytosol (3), it lacks a mechanism to acquire extracellular fMet.

Translation is an energetically costly process, and thus regulation of initiation is coupled to nutrient availability via one-carbon metabolism and the folate cycle (5). Decoupling of translation and the energy status of the cell generally lead to hypersensitivity to stress conditions. Feng et al. showed that fMet derived from THF metabolism is critical for *Lm* virulence, specifically in the livers of infected mice. Although this organ-specific phenotype cannot yet be mechanistically explained, it suggests that specific stressors encountered by *Lm* in the liver require robust regulation of translation to produce specific stress-relieving or virulence-associated proteins. Indeed, *S. aureus fmt* mutants are substantially less pathogenic at least partially due to a decrease in secreted proteins, including virulence factors such as α-hemolysin and coagulase (6). Additionally, Feng et al. demonstrate that a purine auxotrophic strain is attenuated in the livers but not the spleens of infected animals. These results suggest that *Lm* may have differential access to purines *in vivo*, potentially contributing to the more dramatic defect of the 10-formyl-THF-deficient mutant in the liver.

fMet is important for translation initiation in all prokaryotes and is, therefore, sensed by pattern recognition receptors on innate immune cells. In mice, formylated peptide receptors Fpr1 and Fpr2 detect formylated peptides produced by *Lm* and are required for neutrophil chemotaxis to the site of infection and ultimately *Lm* clearance (7). The results from Feng et al. reveal that one consequence of using antifolate antibiotics to treat listeriosis is a decrease in formylated peptide production, which would decrease neutrophil chemotaxis and innate immune-mediated elimination of *Lm*. Currently, known resistance mechanisms to antifolates depend on mutations to the drug target enzyme, which render the folate pathway fully functional (8). However, if *Lm* could develop an alternative antifolate resistance strategy, it would then become a stealth invader, essentially invisible to the innate immune system.

Taken together, the comprehensive study of *Lm* folate metabolism by Feng et al. revealed that *Lm* can obtain dTMP, purines, and most amino acids from the host environment, but fMet biosynthesis is crucial for the intracellular growth and virulence of *Lm. Lm* mutants lacking fMet exhibited organ-specific growth defects, likely due to differential nutrient availability and varying exposure to stresses. This study deepens our understanding of *Lm* pathogenesis and provides valuable insights for optimizing the use of antifolate antibiotics.
